# Indocyanine Green for Assessment of Ureteral Vascularity Can Reduce the Rate of Ureteral Complications in Patients Undergoing Extended Hysterectomy

**DOI:** 10.3390/jcm13185425

**Published:** 2024-09-13

**Authors:** Maja Mrugała, Marek Fiutowski, Krzysztof Nowak, Zofia Borowiec, Mariusz Kasperski, Wiktor Bek, Aneta Machnicka-Rusek, Ewa Milnerowicz-Nabzdyk

**Affiliations:** Clinical Department of Oncological Gynecology, University of Opole, 45-401 Opole, Poland

**Keywords:** hysterectomy, ureteral vascularity, indocyanine green, ureteral complications, deep endometriosis, double-J stenting

## Abstract

**Objectives**: This study aimed to evaluate the effectiveness of using indocyanine green (ICG) for assessing ureteral vascularity to reduce ureteral complications in patients undergoing extended hysterectomy for deep endometriosis or oncological indications. **Methods**: A retrospective-prospective cohort study was conducted at the Centre of Gynecology in Opole, Poland, involving 555 patients who underwent hysterectomy from 2020 to 2023. Patients were categorized based on the Querleu–Morrow classification. ICG was used intraoperatively for vascular assessment in patients with deep endometriosis undergoing wide ureter dissection typical of Type C hysterectomy. **Results**: Ureteral complications occurred in 12 (2.2%) patients, with a significantly lower complication rate in those who underwent ICG testing (1.7%) compared to those who did not (22.7%, *p* = 0.001). Prophylactic double-J stenting further reduced the risk of complications. **Conclusions**: The use of ICG for intraoperative assessment of ureteral vascularity significantly reduces the risk of ureteral complications in complex hysterectomies. Further studies are needed to confirm these findings.

## 1. Introduction

### 1.1. Definition of Endometriosis and Classification of Hysterectomies

Endometriosis is a condition where endometrial-like tissue grows outside the uterus, causing severe pelvic pain and potential infertility. It can begin with menarche and persist until menopause, leading to inflammation and scarring in the pelvis and, rarely, outside the pelvis. Types include superficial pelvic peritoneal lesions, ovarian cysts (endometriomas), and deep lesions in the recto-vaginal septum, bladder, and bowel. Endometriosis affects approximately 10% (190 million) of reproductive-age women and girls globally. The cause of endometriosis is poorly understood, with no prevention or cure, but symptoms can be managed with medication or surgery [1]. Classifying endometriotic lesions is essential for evaluating the disease’s extent, anatomical location, and clinicopathological impact and for guiding treatment planning. The #Enzian classification is a universally usable system based on division into three compartments (A—vagina, rectovaginal space (RVS); B—uterosacral ligaments (USL)/cardinal ligaments/pelvic sidewall and C—rectum). The severity grade is determined by the maximum diameter of the endometriotic lesion [2]. Deep endometriosis is defined as the occurrence of subperitoneal infiltrations with a depth of more than 5 mm [2,3].

Surgical treatment for endometriosis is recommended when conservative treatment and lifestyle changes have not effectively managed the symptoms or in the occurrence of severe pain, infertility, or deep endometriosis. In women with severe deep endometriosis, a hysterectomy combined with the removal of endometriotic lesions, with or without oophorectomy, can be considered. However, this decision should align with the patient’s family planning goals [3].

In contemporary cervical cancer surgery, tailoring surgical radicality is paramount to optimizing patient outcomes. Considering the extent of parametrial involvement in surgery, Querleu and Morrow developed a classification system for hysterectomy. They categorized the procedure into four distinct types, each defined by the degree of lateral resection and surgical radicality. Type A (limited radical hysterectomy) involves minimal mobilization of the ureter and limited resection of paracervical tissues, thus posing a relatively low risk of ureteral injury. In this type, the ureter is only minimally dissected to ensure its preservation during the excision of the cervix and adjacent structures. Type B (modified radical hysterectomy) requires more extensive dissection of the ureter. The resection encompasses the paracervix at the level of the ureter, necessitating meticulous mobilization and safeguarding of the ureter. This procedure is indicated when more extensive tissue removal is required while still aiming to maintain ureteral integrity. Type C, referred to as radical hysterectomy, involves the most comprehensive ureteral dissection. The procedure includes transecting the paracervix at its junction with the internal iliac vascular system, necessitating complete mobilization of the ureter from surrounding tissues. This approach is essential for achieving radical excision of malignant lesions with clear margins and addressing potential nodal metastasis sites. Ureteral management in Type C hysterectomy is critical, as the extensive dissection significantly elevates the risk of ureteral injury. Type D hysterectomy involves ultra-radical procedures where structures lateral to the paracervix are resected, including the entire paracervix and adjacent vessels, exposing the roots of the sciatic nerve expanding to adjacent fascial and muscular structures and is typically performed for laterally recurrent tumors [4,5].

In benign gynecological surgery, a hysterectomy may occasionally necessitate the incorporation of radical surgical techniques. This is particularly relevant in cases involving extensive adhesions, large fibroid uteri, or deep endometriosis. These complex conditions often require a more comprehensive surgical approach to ensure complete removal and achieve optimal patient outcomes.

### 1.2. Hysterectomy and Complications Related to the Urinary System

Hysterectomy performed for deep endometriosis carries an increased risk of perioperative and postoperative complications. This risk is estimated to be four times higher than in hysterectomies performed for other reasons and includes hemorrhage and direct injuries to the bowel, bladder, and ureter due to challenging adhesiolysis [3]. Ureteral complications investigated in this study are common in obstetric and gynecologic surgeries. It is estimated that ureteral injuries occur in approximately 0.2–1% of all pelvic surgeries and up to 30% of radical hysterectomies. Importantly, half of all ureteral injuries are associated with obstetric and gynecological surgeries. Types of ureteric injuries include crushing, ligation, transection, angulation, excision, ischemia from stripping, electrocoagulation, and resection, either alone or in combination. Depending on their severity and how they are managed, these injuries can lead to the development of stenosis or the formation of a ureterovaginal fistula [6]. Ureteral leaks can potentially be overlooked during surgery, resulting in the development of an iatrogenic ureterovaginal fistula and persistent urinary leakage from the vagina [7,8,9]. The formation of a uretero-abdominal fistula leads to fluid collection in the abdominal cavity, particularly in the Douglas pouch, and infection [10]. The development of stenosis can potentially lead to hydronephrosis and renal failure [11]. An additional concern in case of complications is the requirement for surgical intervention following hysterectomy and ureter stenting. 

### 1.3. Injuries to the Urinary System Increase Morbidity; Thus, Methods Are Sought to Minimize This Risk

The high risk of ureteral injuries during obstetric and gynecological surgeries increases morbidity and treatment costs, prompting the search for methods to minimize this risk. Ureters may be injured during hysterectomy due to their proximity to the uterus and anatomical variations. These variations can include duplication, retroiliac positioning, and ureteric diverticula. Although anatomical variations of the ureter have not been extensively studied, they must be considered during surgical procedures [12]. One method to identify ureters and reduce ureteral injury during complex surgical procedures involving hysterectomy is the use of indocyanine green (ICG) [13,14]. Data from the literature suggest that this new concept can enhance the assessment of ureteral vascularity impairment during minimally invasive abdominal operations, with increasing evidence supporting its effectiveness [15,16].

There are two approaches to using ICG. One option is to administer ICG intraureterally via ureteric cannulation, allowing for precise real-time visualization of the ureters by the near-infrared camera system to assess the exact position of the ureter during extensive surgeries [17]. The other requires an intravenous injection of ICG to evaluate the vascularity of ureters to support surgical decisions [18]. It is worth noting that intravenous administration of ICG has not been approved in some countries, leaving such use off-label; for this reason, intraureteral administration remains the only choice [19]. A systematic review of the literature on the use of ICG during minimally invasive procedures for treating benign gynecologic pathologies found that ICG was safe and no complications after its administration were reported [20]. In our institution, the second approach of using ICG for the assessment of ureter vascularity visualization is used. Nevertheless, further experience is required to confidently integrate the use of ICG in laparoscopic surgeries into clinical practice. This study addresses this gap and presents a single-center experience using ICG during type C laparoscopic hysterectomies. 

## 2. Objective

In this study, we presented the outcomes of extended hysterectomy procedures performed in our institution. The objective was to determine the frequency of adverse events following different types of hysterectomy. In addition to this aim, we hypothesized that using ICG to assess the vascularity of the ureter during a minimally invasive laparoscopic technique can reduce the rate of ureteral adverse events in complex surgeries involving hysterectomies. In the case of reduced perfusion after the dissection of the ureters, we explored whether prophylactic stenting can further reduce complication rates. 

## 3. Methods

### 3.1. Design

A retrospective-prospective cohort study was conducted to present the characteristics of patients undergoing radical hysterectomy at the Centre of Gynecology in Opole, Poland. All these patients consented to hysterectomy due to deep endometriosis and/or adenomyosis, completed their reproductive plans, and wished to minimize the risk of recurrence of deep endometriosis. The study included consecutive patients treated between 2020 and 2023.

### 3.2. Study Group

Patients who had complete data on the diagnosis, type of surgical procedure, and postoperative course with description of complications were included. In hysterectomy types A and B, patients underwent surgery either through laparotomy or using the minimally invasive laparoscopic approach based on shared decision-making with the patient. All patients undergoing extended hysterectomy with ureteral dissection for deep endometriosis were operated on using a minimally invasive technique with ICG to assess ureteral vascularity. The oncological procedures were performed using either the open technique or the minimally invasive technique but without assessing ureteral perfusion with ICG. The ICG technique used in extended hysterectomy for deep endometriosis was employed to detect cases with the highest risk of ureteral complications. Ureteral complications considered in this study included ureteral leaks, all types of fistulas, and ureteral stenoses.

### 3.3. Procedure

All patients underwent hysterectomies classified according to the Querleu–Morrow classification into three types: A, B, and C. Type D hysterectomy was not performed [4,5].

It is crucial to admit that, for the purposes of this study, to standardize the assessment of our procedures for ureter protection, we established a conventional division of patients undergoing hysterectomy based on the Querleu–Morrow classification, with specific reference to ureter dissection procedures. This adjustment was made because not all patients required the full extent of the surgery as defined in the original classification due to their non-oncological diagnosis, such as deep endometriosis. In our center, nearly all of the patients suffering from deep endometriosis included in the study underwent comprehensive surgery with concurrent dissection of the pararectal fossae and, in some cases, also the paravesical fossae. In all cases, parametrial dissection was performed. The extent of ureteral dissection largely corresponded to Type C radical hysterectomy.

All patients with deep endometriosis were qualified for surgery after a detailed anamnesis, gynecological examination, pelvic MRI, and a comprehensive ultrasound evaluation of the pelvic organs and kidneys. This approach allowed for precise determination of the scope of the surgery and thorough preparation of both the patient and the surgeon. The procedures were performed at a center in Poland with the highest referral level for deep endometriosis by a specialized team consisting of a lead expert surgeon and four trained assistants, ensuring consistency in the quality and method of the surgeries. In our center, mostly complex procedures are performed, with concomitant bowel surgery being performed in 89% of cases, thus necessitating frequent ureter dissection, opening of the Latzko and Okabayashi spaces, and the assessment of bowel anastomosis and ureteral vascularity using ICG. In our group, 20% of the operated patients had deep endometriosis affecting the bladder, while 13% had ureteral infiltrations requiring procedures other than ureterolysis, such as shaving, ureter reimplantation, or nephrectomy.

**Figure 1:**
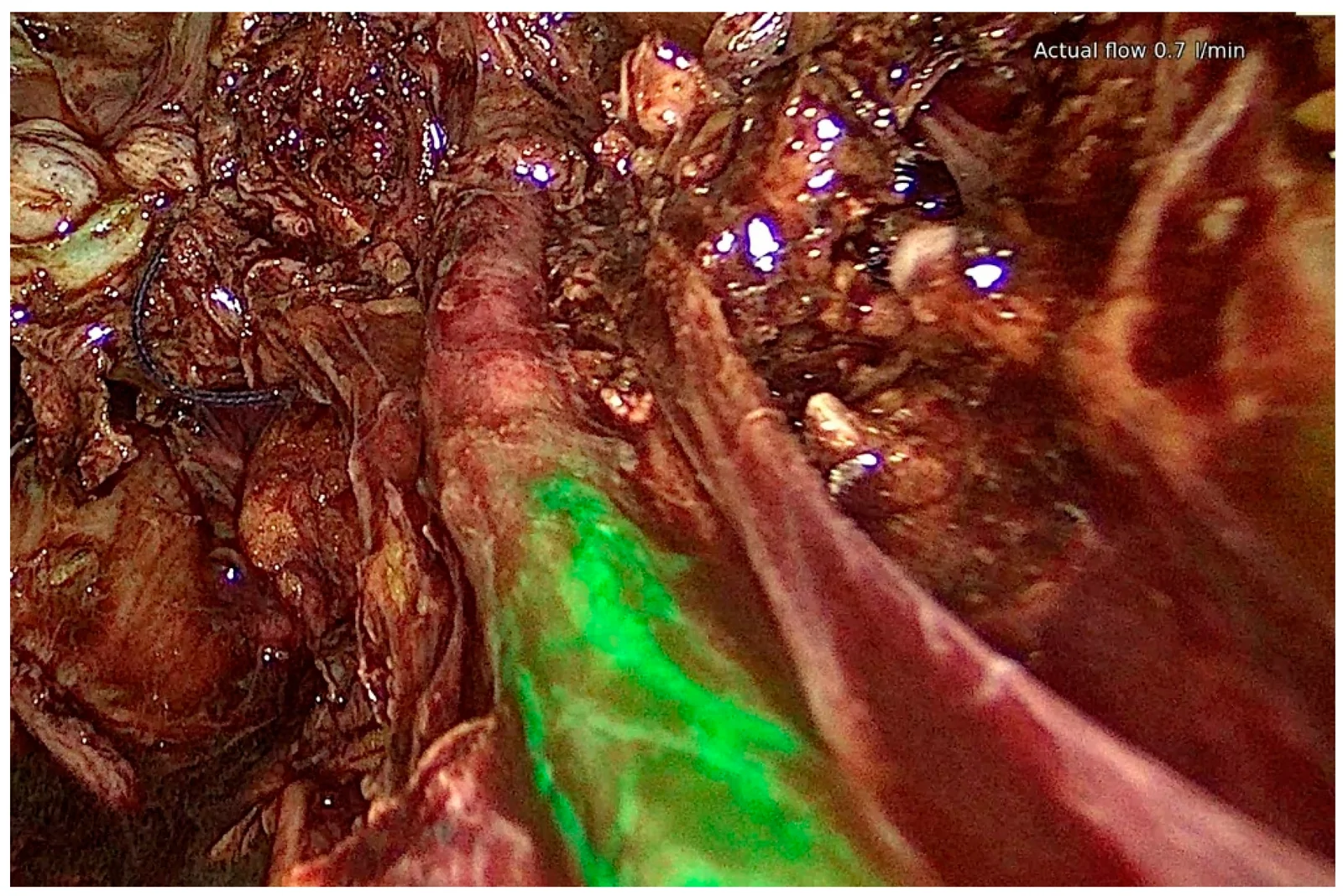
Laparoscopic image taken with the ICG camera showing the evaluation of ureteral vascularity—the green color shows the presence of indocyanine green dye.

The surgery was conducted either with laparotomy or laparoscopy. Laparotomy is recommended by current guidelines for oncological cases, such as ovarian cancer with peritoneal dissemination or cervical cancer. Patients with advanced deep endometriosis undergo laparoscopic surgery, as recommended for non-oncological diseases; however, the surgical scope in these cases is comparable or broader than in oncology cases. The ICG testing was used in patients with deep endometriosis operated with the minimally invasive approach. The vascular evaluation of the ureters was performed after ureteral preparation to detect any injuries and assess the associated risks. We have used VERDYE (Diagnostic Green, Athlone, Ireland) [21,22]. According to the prescribing information, one dose per measurement for adults, elderly individuals, and children for diagnosing heart, circulatory system, microcirculation, tissue perfusion, and cerebral blood flow is 0.1 to 0.3 mg/kg body weight, administered as a bolus injection. In our institution, we administer half an ampoule, which is 12.5 mg in 2.5 mL of injection water. The VERDYE solution was administered after the completion of the surgical procedures. A few seconds post-administration, we assessed the uniform network of vessels along the entire segment of the dissected ureters using the ICG camera (with an infrared module). The ICG flow in the vessels supplying the ureter was assessed visually. We evaluated the uniform network of vessels along the entire segment of the dissected ureters. The ureteric vascularity image is shown in Figure 1. 

Apart from dividing patients based on the Querleu–Morrow classification, patients undergoing type C hysterectomy were further divided into those who underwent laparoscopic hysterectomy for deep endometriosis with ICG testing (C ICG) and those who underwent hysterectomy C for deep endometriosis or cancer and were not tested with ICG (C non-ICG). 

In patients with impaired vascularity, defined as reduced or zero flow in any segment of the ureter, a decision was made to perform a cystoscopy and place a double-J (DJ) ureteral stent (Pigtail 4.8F/26, Balton sp. z o.o., Warsaw, Poland) under the guidance of a laparoscopic camera as a prophylactic measure to reduce the risk of ureteral complications. The DJ stents were inserted post-surgery when ICG testing indicated impaired ureteral vascularity. In our institution, this method is employed to prevent the development of ureterovaginal fistulas, peritoneal leaks, and strictures. Therefore, when anticipating a higher risk of these complications in a poorly vascularized ureter, we perform conventional DJ ureteral stenting. 

A DJ stent is placed for 12 weeks to secure the patient until potential repair in case of stenosis after stent removal. This approach is based on patient preferences—each patient has the option to leave the stent for the standard duration of six weeks, with the risk of needing a nephrostomy if removed sooner in case of complication, or to extend it to 12 weeks. The 12-week time of stent retention, established in collaboration with urologists, was deemed optimal for potential repairs of delayed ureteral complications, allowing intervention after 12 weeks. After 12 weeks, the stents were removed during cystoscopy. The increased risk of ureteral injury was defined by impaired ureteral vascularity visualized with ICG. The same operators were involved in assessing the validity of stent placement. This comprehensive approach ensures that patients clearly understand their options and the associated risks, enabling them to make informed decisions about their care. Additionally, this method aims to minimize the need for emergency interventions and maximize the chances of successful long-term outcomes for patients with significant ureteral risk factors.

### 3.4. Statistical Analysis

Statistical analysis was conducted using the R Project for Statistical Computing v. 3.4.1. Categorical data were presented as numbers and percentages, while continuous data were presented as means and standard deviation. Categorical data were compared using tables contingency tables and the Fisher exact test. The differences were considered statistically significant at a *p*-value of <0.05.

## 4. Results

In total, 555 patients were included in the study, of which 301 (54.2%) underwent type A hysterectomy, 59 (10.6%) underwent type B hysterectomy, and 195 (35.1%) underwent type C hysterectomy (based on the aforementioned division regarding ureteral dissection). The mean age of the included patients was 55.88 ± 14.55 years. The urinary system complications included stenoses and fistulas; their rate was 2.2%. The characteristics of the study group are presented in Table 1.

Ureteral complications occurred in 12 (2.2%) women. There were 5 (0.9%) cases of stenosis and 7 (1.3%) cases of fistula. When analyzing complications by type of hysterectomy in the total study group, 1 (0.2% of total number complications) case was reported in a patient undergoing type A hysterectomy, 3 (0.5%) cases were reported in patients undergoing type B hysterectomy, and 8 (1.4%) cases were reported in patients undergoing type C hysterectomy. It can be noted that the proportion of complicated cases gradually increased with the advancing type of hysterectomy. 

Among patients undergoing type C hysterectomy for deep endometriosis, those operated on using laparoscopic techniques with ICG testing had significantly fewer complications compared to those who underwent laparotomy without ICG (1.7% vs. 22.7%; *p* = 001). The difference between groups was statistically significant. The comparison of complication rates according to type of hysterectomy is outlined in Table 2.

DJ stenting was used in 98 patients (17.7% of the entire study group) who were considered at high risk of ureteric complications, as assessed by the operator, with the aim of maintaining the stents for 6–12 weeks. However, poor tolerance of the stents was observed in 2 patients, necessitating their removal before this 3-month period was completed.

The use of two protective measures against ureteric complications involving the identification of a high-risk group with the ICG test followed by ureteral stenting with DJ stent allowed for the elimination of complications such as leaks. The only complications in this group were ureteral stenoses, which were electively managed through ureteral reimplantation after three months (1.1%). Complications such as fistulas occurred in one patient (0.6%) from the group in which the ICG test was performed but without subsequent stent placement. In contrast, in patients who did not undergo the ICG test, fistula formation was detected in 3 (13.6%) patients with a *p*-value of 0.005. The rates of complications by type in the group of patients undergoing type C hysterectomy are shown in Table 3.

## 5. Discussion

In our study, ureteral complications occurred in 12 (2.2%) women in 555 patients. The percentage of ureteral complications increased with the complexity of the hysterectomy procedure. It was 0.3% for type A, 5.1% for type B, and 4.1% for type C (*p* = 0.002) but reached 22.7% among women who underwent hysterectomy C for oncological reasons or deep endometriosis and were not tested with ICG. The rate of ureteral complications among patients undergoing type C hysterectomy with ICG testing was significantly lower in comparison to those without ICG testing (1.7% vs. 22.7%; *p* = 0.001). Adjunctive use of DJ stenting following ICG testing for 12 weeks eliminated fistulas and decreased the rate of stenoses in this high-risk group.

The rate of ureteral complications in our institution ranged from 0.7% to 22.7%, depending on the complexity of the surgical procedure. Other researchers have reported complications involving the ureter following hysterectomy; however, the rates of ureteric complications vary among studies due to methodological differences, including eligibility criteria for the populations studied. There is little evidence of post-surgical ureteral complications. The estimated rate of cumulative surgical complications for patients treated for ureteral endometriosis reaches 9%, including recurrent ureteral obstruction (7.4%) and ureteral or ureterovaginal fistula (1.6%) [23]. Ureteric injuries occurring during hysterectomies in Norway were analyzed by Ravlo et al. [24]. Their retrospective study, based on data from the Norwegian Patient Registry, covered a period of 11 years during which 53,096 hysterectomies were registered. Overall, ureteric injuries were reported in 643 (1.2%) women. Symptoms indicative of ureteric complications included pain (77%), fever (12%), urinary leakage (13%), and anuria (8%). Up to 77% of women experiencing ureteric complications required surgical intervention. Furthermore, 10% of women with these complications lost a kidney, underscoring the importance of implementing measures to diagnose ureteric injuries during surgery or as quickly as possible afterward. A meta-analysis conducted by Yanagisawa et al. [25] demonstrated that laparoscopic hysterectomy, in contrast to laparotomy, carries a higher risk of ureteric injuries, with a pooled odds ratio (OR) of 2.12 (95% confidence interval (CI), 1.71–2.62). The authors also found that early identification of ureteric complications during surgery is associated with a lower rate of management failure compared to detection after surgery (pooled OR 0.22, 95% CI 0.12–0.41). Dallas et al. [26] analyzed a large dataset of US women who were subjected to hysterectomy for benign indications between 2005 and 2011. In total, 296,130 women were included, of which 2817 (1.0%) experienced ureteral injuries and 831 (0.3%) women developed a fistula. Park et al. [27] conducted a retrospective study covering 2927 women who underwent any type of gynecologic surgery over five years between 2006 and 2011. Ureteral injuries were reported in 35 patients (1.2%). Of all those cases, in 20 patients, ureteral injuries were identified during surgery, and in 15 patients, up to 28 days after surgery. The authors reported that the frequency of late detection of ureteral injuries was higher for minimally invasive procedures than for laparotomy (73% vs. 29%; *p* = 0.027).

In our study, none of the patients undergoing hysterectomy experienced any serious ureteral complications leading to loss of the kidney. However, many researchers report that such complications are possible, particularly when ureteric injuries are overlooked after surgery. Ravlo et al. [24] reported that 10% of women with ureteric complications lost a kidney. Sagayanathan et al. [28] described a case of a woman with a history of hysterectomy due to endometriosis who developed hydronephrosis and hydroureter. The authors highlight that ureteric dilations and obstructions occurring long after surgery can also result from soft tissue lesions caused by ureteric endometriosis. Youssef et al. [29] also confirmed the contribution of urinary tract endometriosis in patients with and without a history of hysterectomy to the development of asymptomatic silent obstructive uropathy and ultimately to kidney failure. 

Research shows that it is essential to identify ureteric injury early during surgery, as prompt repair reduces the risk of subsequent serious complications. Park et al. [27] reported that all ureteral injuries detected at the time of surgery were repaired with satisfactory results. On the other hand, only two ureteral injuries diagnosed up to one month after surgery underwent a reparative surgical procedure, while others were treated with different types of stenting. The study conducted by Dallas et al. [26] demonstrated that fistula formation was less frequent when the injury was noted during the hysterectomy compared to being discovered later in the perioperative period (0.7% vs. 3.4%, *p* < 0.001). Furthermore, prophylactic ureteric stenting reduces the frequency of ureteric injuries (pooled OR 0.61, 95% CI 0.39–0.96) [26]. A meta-analysis conducted by Yanagisawa et al. [25] produced similar findings, showing that detecting a ureteric injury at the time of surgery resulted in a lower risk of complication management failure in comparison to identifying it after surgery (pooled OR 0.22, 95% CI 0.12–0.41). 

Due to the advantages of early detection of ureteral injuries, several methods have been proposed and used to reduce the rate of ureteric complications of gynecological operations. The most commonly employed options include measures that allow intraoperative detection of ureteric injuries, such as ICG testing and ureteric stenting. ICG testing can improve the intraoperative evaluation of organ vascularity and support the surgeon’s intraoperative decision-making, while ureteral stenting supports healing. Our study proved that using both procedures significantly reduced the frequency of ureteral complications. To our best knowledge, a combination of both procedures in patients undergoing radical hysterectomy has not been published. In patients with ureteral endometriosis, perfusion assessment with ICG was used to detect local ischemia and support decision-making regarding ureteral stent placement. In this preliminary study, Raimondo et al. [18] evaluated 31 ureters intraoperatively with ICG imaging. In 5 cases (16.1%), Notably, this study reported a high rate of interoperator agreement in classifying residual ureteral perfusion during ICG testing. Additionally, no intraoperative complications, adverse effects from ICG use, or post-ureteral procedure leakage were reported. Kanno et al. [30] described a case of a woman suffering from severe chronic pelvic pain and dysmenorrhea. She was diagnosed with uterine adenomyosis and left ovarian endometrioma with deep endometriosis of the uterosacral ligament, posterior cervix, and rectum. Although the ICG testing was not specifically targeted at the ureters, it helped limit the extent of surgery and enabled a nerve-sparing operation for extensive deep endometriosis. The postoperative course was uneventful. After the successful use of ICG dye in laparoscopic surgeries, there have been attempts to use other agents for the same purpose. In a recent study conducted by Farman et al. [31], a new dye was tested during minimally invasive hysterectomy in 24 women. This phase 1 study aimed to evaluate the safety of the new agent, but among the secondary endpoints was the ease of identification of ureters during gynecologic surgeries. The intensity of ureteral fluorescence was dose-dependent.

### Limitations

Several limitations of this study have to be considered when interpreting the results. First, this is a retrospective-prospective study in which some participants were selected from the existing medical records, and the study population does not represent the entire population. The availability and accuracy of historical data are usually lower in comparison to prospective data collection, which can potentially lead to limited control for confounding factors. Moreover, patients undergoing laparotomy differed from those undergoing laparoscopic surgery in terms of diagnosis. All these features can impact the study’s outcomes. Second, ureteral complications during or after hysterectomy are rare, which can potentially result in the study being underpowered. However, the literature review conducted to look at the incidence of ureteral complications at other institutions showed that our complication rates are similar. Finally, the involvement of several operators introduces variability that cannot be excluded. To reduce this variability, the same operators assessed the validity of using stents. 

## 6. Conclusions

Type C dissection ureter surgery and multi-organ surgeries in deep endometriosis involving extended hysterectomy are at increased risk of ureteral complications. Using ICG to identify the ureter injury during such complex surgeries can improve the outcomes of minimally invasive laparoscopic treatment. It can also facilitate decision-making in choosing less invasive procedures. Our preliminary findings showed the usefulness of ICG testing; however, further prospective studies on larger populations are needed to verify these findings.

## Figures and Tables

**Table 1:** Characteristics of the study group.

Characteristic	Sub-Group	Number (%)
Diagnosis	Cervical cancer	28 (5.0)
	Ovarian cancer	94 (16.9)
	Endometrial cancer	260 (46.7)
	Deep endometriosis	175 (31.4)
Operation	Laparoscopy	375 (67.4)
	Laparotomy	174 (31.4)
	Conversion to laparotomy	7 (1.3)
Complications	Stenosis	7 (1.3)
	Fistula	5 (0.9)
	Uncomplicated course	543 (97.8)

**Table 2:** Rates of complications in patients undergoing radical hysterectomy by type of procedure.

Hysterectomy Type	n (%)	Complications n (%Grade Group)	*p*-Value
A	301 (54.2)	Yes—1 (0.3) No—300 (99.7)	0.002
B	59 (10.6)	Yes—3 (5.1) No—56 (94.9)	
C	195 (35.2)	Yes—8 (4.1) No—187 (95.9)	
C ICG	173 (31.2)	Yes—3 (1.7) No—170 (98.3)	0.001
C non-ICG	22 (4.0)	Yes—5 (22.7) No—17 (77.3)	

C ICG: women undergoing type C hysterectomy for deep endometriosis with a minimally invasive procedure and ICG testing; C non-ICG: women who underwent hysterectomy C for deep endometriosis or cancer and were not tested with ICG; ICG: indocyanine green.

**Table 3:** Rates of complications in patients undergoing type C endometriosis.

Type of Ureteric Complication	Type C Hysterectomy	n (%)	Complications n (%Grade Group)	*p*-Value
Fistula	C ICG	173 (88.7)	Yes—1 (0.6) No—172 (99.4)	0.005
	C non-ICG	22 (11.3)	Yes—3 (13.6) No—19 (86.3)	
Stenosis	C ICG	173 (88.7)	Yes—2 (1.2) No—171 (98.8)	0.064
	C non-ICG	22 (11.3)	Yes—2 (9.1) No—20 (90.9)	

C ICG: women undergoing type C hysterectomy for deep endometriosis with a minimally invasive procedure and ICG testing; C non-ICG: women who underwent hysterectomy C for deep endometriosis or cancer and were not tested with ICG; ICG: indocyanine green.

## Data Availability

Dataset available on request from the corresponding author.
